# Immunotherapeutic Potential of Oncolytic Vaccinia Virus

**DOI:** 10.3389/fonc.2014.00155

**Published:** 2014-06-17

**Authors:** Steve H. Thorne

**Affiliations:** ^1^Department of Surgery, Hillman Cancer Center, University of Pittsburgh Cancer Institute, University of Pittsburgh, Pittsburgh, PA, USA; ^2^Department of Immunology, Hillman Cancer Center, University of Pittsburgh Cancer Institute, University of Pittsburgh, Pittsburgh, PA, USA

**Keywords:** oncolytic virus, cytokine, chemokine, vaccine, MDSC

## Abstract

The concept of oncolytic viral therapy was based on the hypothesis that engineering tumor-selectivity into the replication potential of viruses would permit direct destruction of tumor cells as a result of viral-mediated lysis, resulting in amplification of the therapy exclusively within the tumor environment. The immune response raised by the virus was not only considered to be necessary for the safety of the approach, but also something of a hindrance to optimal therapeutic activity and repeat dosing. However, the pre-clinical and subsequent clinical success of several oncolytic viruses expressing selected cytokines has demonstrated the potential for harnessing the immune response as an additional and beneficial mechanism of therapeutic activity within the platform. Over the last few years, a variety of novel approaches have been incorporated to try to enhance this immunotherapeutic activity. Several innovative and subtle approaches have moved far beyond the expression of a single cytokine transgene, with the hope of optimizing anti-tumor immunity while having minimal detrimental impact on viral oncolytic activity.

## Background

Viral infections and cancer bear a variety of striking similarities, as seen with the fact that several cancers are caused as a result of chronic viral infection (1, 2) and the fact that the first oncogenes were identified through their homology to viral genes (3, 4). Indeed, many of the hallmarks of cancer strongly resemble the adaptations a virus induces in a susceptible cell during its replication cycle (5). It is therefore unsurprising that some viral virulence genes are redundant for replication in malignant cells or the tumor microenvironment, meaning that their deletion results in the production of vectors whose replication is attenuated in normal, but not cancer cells (6). This finding was the basis for the design and construction of the first oncolytic viral therapies (7, 8). As the name “Oncolytic” suggests, these were hypothesized to function through the direct destruction of cancer cells, primarily as a result of viral replication in infected cells, but also as a result of immune recognition of these cells. Initial clinical testing of this approach centered around strains of adenovirus (8–12), perhaps more due to the historical use of non-replicating adenoviruses as gene therapy vectors than because of any special attributes of this backbone particularly appropriate for an oncolytic platform. However, importantly these early clinical studies demonstrated both safety and therapeutic responses (13–16). The observation that the viral infection occurring primarily within the tumor was cleared, leading to induction of anti-viral immunity, and implied the agents were capable of at least transiently overcoming localized immune suppression within the tumor.

The slow replication and limited systemic spread of the Ad5-based vectors proved to be especial limitations (17, 18), however, the excellent safety profile and indications of responses led investigators to examine other adenoviral serotypes and more potent viral backbones as the basis for next generation oncolytics. In some cases, combination with immunosuppression was investigated as a means to enhance oncolytic activity through delaying clearance of the therapy (19, 20). However, in general pre-clinical and clinical testing of different viral backbones in combination with expression of different therapeutic transgenes led to the observation that the most effective approaches frequently incorporated rapidly replicating viral backbones expressing cytokines as transgenes, notably GM-CSF (2, 21–23). In addition, when immunocompetent pre-clinical tumor models were available, it was frequently seen that complete responses after viral therapy was coupled with induction of anti-tumor immunity and the capacity to reject re-challenge with the same tumor cell lines (24). As such considerable focus has turned to development of approaches to enhance or optimize this immunotherapeutic effect. However, there is clearly a fine balance to be considered as robust induction of the immune response can lead to premature clearance of the therapy, meaning that the oncolytic effects are lost and adaptive immunity is targeted against the viral component only, with little or no cross-presentation of tumor antigens.

One viral strain that has been developed as an alternative to adenovirus in forming the backbone of many oncolytic viral strains is vaccinia virus (25). This enveloped DNA virus has a large genome, with many virulence genes that target host cell cycle, apoptotic pathways, or immune response, and whose deletion leads to viral strains with demonstrated tumor-selective replication (26–28). In addition, the use of this virus during the eradication of smallpox means that its immune activating capacity is well understood. The clinical use of a GM-CSF expressing oncolytic vaccinia virus has also been instrumental in demonstrating the potential for enhancing the immune response induced by oncolytic viruses as a means to enhance therapeutic activity (23, 29, 30). Oncolytic vaccinia will therefore be used as the primary example to illustrate the potential for enhancing immune-mediated mechanisms in this platform throughout the remainder of this review.

## Approaches to Enhance the Anti-Tumor Immune Response Induced by Oncolytic Vaccinia

Several standard strategies have been routinely applied to enhance the anti-tumor immune response induced through oncolytic viral therapy, primarily focused on expression of either cytokines or co-stimulatory molecules (31). Although several vaccinia and related vectors expressing single cytokines have clearly demonstrated therapeutic benefits in both animal models and in the clinic, this approach typically suffers from several handicaps, including reduced viral replication due to reduced initial infection of the tumor or early clearance of the virus (32); in addition, the use of a single cytokine means that typically only one step in the complex kinetic process of innate to adaptive immune response can be stimulated. The most clinically successful approach to date has been the expression of GM-CSF (33), both from vaccinia virus and from oncolytic HSV. The choice of GM-CSF was based on early reports that expression of GM-CSF from mouse melanoma cell lines resulted in failure of these cells to form tumors in syngeneic mice (34). It is likely that the reason for the success of vectors expressing this cytokine is primarily based on the fact that it has broad effects on induction of proliferation in many immune cell subtypes, while having little or no direct anti-viral properties. However, more recently the role of GM-CSF expression in increasing proliferation of some suppressive cells [notably monocyte derived suppressor cells (MDSC)] (35) has been elucidated meaning that some caution in the use of this cytokine might be needed.

Several other cytokines, including IL-2, TNF, and IFN have been used in pre-clinical vaccinia-based models but have not been successfully translated into a clinical setting, possibly due to their capacity to also induce more directly anti-viral effects (32, 36, 37). Because of this limited success with cytokines other than GM-CSF alternative immune stimulating strategies has been explored.

For example, the expression of antibodies represents a promising strategy. The relative success of monoclonal antibody therapy and the recent emergence of antibodies targeting immune checkpoint inhibitors or that mimic co-stimulators has demonstrated the potential of this platform. The requirements for expression and assembly of multiple large peptides had traditionally limited the use of antibodies as transgenes, however, more recent development of single peptide antibodies means this is likely to be a fruitful approach moving forward and initial reports of vaccinia strains expressing antibodies are promising (38).

An alternative strategy to enhancing the immune effects of oncolytic viruses is to express chemokines from the vectors that specifically attract T-cells into the tumor (39, 40). This approach appears to have minimal negative impacts on viral replication and oncolytic activity, yet enhances the immunotherapeutic effects. One of the major hurdles found with the use of therapeutic tumor vaccines has been the limited trafficking of tumor-specific T-cells into the tumor itself, so the combination of chemokine expressing oncolytic viruses with vaccination against tumor antigens may be a promising strategy.

**Figure 1:**
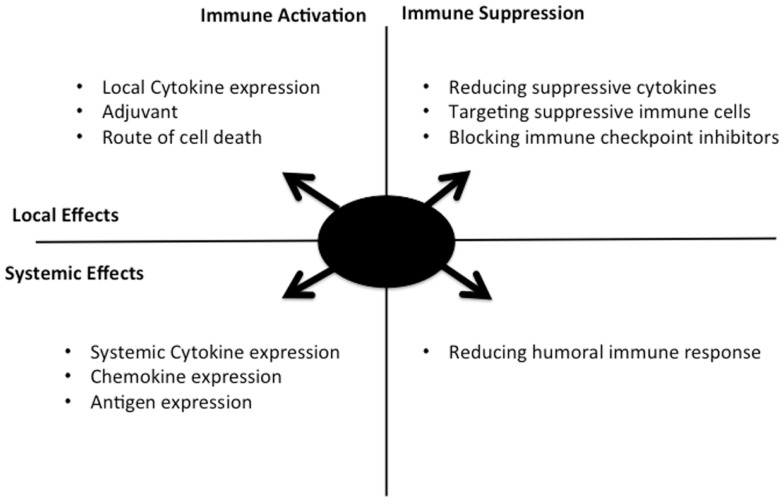
**Selective replication of an oncolytic virus within an infected tumor cell might be engineered in multiple ways to optimize the kinetics, type, and level of resultant immune response**. The approaches covered in this perspective are summarized here, along with their range of activity (local acting within the tumor, or systemic activity) and whether immune activation or blocking of immune suppression are involved. Ideal combination approaches would be predicted to involve components of different quadrants in the figure.

Several other alternatives to cytokine expression have been explored in different oncolytic backbones as a means to enhance the immunotherapeutic effects (Figure 1), including the following: (i) Enhancing immunogenic cell death, it has been proposed that the route of cancer cell death after therapy may be a critical mediator of the immune response. As a result, adjusting how an oncolytic virus destroys the infected cell may promote a more robust anti-tumor immune response (41). (ii) Targeted inhibition of specific components of the immune response: as an alternative to specifically activating key pathways, key mediators of less desirable immune response pathways may be targeted for removal or depletion. One example is the use of TGFb decoy receptors to remove this cytokine that has been implicated in metastasis and tumor growth and angiogenesis (42). Alternatively, direct targeting of B-cells or other components of the humoral response may limit production of anti-viral neutralizing antibody, a key limiting factor in repeat dosing with the same therapeutic virus (43). (iii) Role of adjuvant: the field of vaccine development has helped define the importance of adjuvant in eliciting a robust immune response. Although the expectation is that the viral vectors themselves will provide a source of potent adjuvant, this can and has been enhanced through expression or manipulation of the virus, such as through the incorporation of CpG rich motifs into the DNA sequence (44), or through combination with CpG (45).

## Alternative Strategies

In addition to the expression of immune stimulatory (or inhibitory) transgenes from the viral vectors, a variety of other options exist that can also have direct impact on the immune responses induced by the viral vectors.

(i) *Viral mutation*: large DNA viruses such as vaccinia or HSV encode multiple virulence genes whose role is to antagonize or deplete specific cytokines or to disrupt steps in the immune response. Selective deletion of specific virulence genes can therefore have two effects; deletion of these genes often results in tumor targeting (through attenuation of viral replication in non-tumor tissues) while also allowing additional induction of specific components of the immune response to be activated (28). In this way, the immune response can be manipulated without seriously depleting viral oncolytic activity.
(ii) *Timing of immune activation*: the therapeutic activity of oncolytic viral therapy can be considered to function in two steps, with an initial directly oncolytic phase mediated by viral lysis of tumor cells followed by an immunotherapeutic phase, where the host immune response clears additional infected tumor cells and ideally results in induction of an adaptive immune response against tumor antigens (as a result of the release of the tumor antigens along with viral PAMPs and host cell DAMPs). It is therefore apparent that the expression of immune activating transgenes would be most effective if limited to this second phase. One approach to achieve this is to apply exogenous regulation of transgene expression or to control the function of the protein after it is translated (32, 37). In this way, it may be possible to achieve unhindered viral replication and so full oncolytic potential during the initial period after viral delivery and tumor infection, while subsequent activation of immune stimulatory transgene activity would enhance the level and type of immune response produced at later times.
(iii) *Targeting immune suppression*: in addition to activating the immune response, oncolytic viral vectors can also transiently overcome immune suppression within the tumor. However, this effect is likely only temporary or limited and some evidence exists that additional targeting of these suppressive cells may further enhance oncolytic viral activity (46). Because multiple suppressive immune cell types (including MDSC, M2 macrophages, and regulatory T-cells) often exist within the tumor, it may be necessary to target all these in a concerted fashion to ensure that a robust adaptive immune response is produced.
(iv) *Combination therapies*: in addition, the development of multiple novel and effective immunotherapies means that the scope for combining oncolytic viral therapies with these other therapies continues to increase. There are a variety of approaches (such as combination with alternative adjuvants or anti-immune checkpoint inhibitors) that would be expected to produce significant synergistic benefit, and promising initial pre-clinical data means that these will be explored in more detail in the future.
(v) *Oncolytic viral vaccines*: the fact that oncolytic viral therapies are capable of inducing an adaptive immune response against tumor antigens is likely to be hugely beneficial in the clearance of residual disease and metastases as well as in long term immune surveillance to prevent relapse. However, it is also likely that any adaptive immune response that is produced after viral therapy will primarily target antigens expressed on the bulk tumor cells. Because the cells that mediate relapse or metastasis often express distinct antigens to those on the bulk tumor cells within the primary tumor, it may be necessary to induce additional immunity against antigens on these cells. It has been demonstrated that the expression of these antigens as peptides or whole proteins from the oncolytic virus can permit additional protection against subsequent relapse (47). It is therefore possible that expression of antigens from the virus may be further used to target other stromal cells within the tumor or to boost the immune response against tumor antigens and away from viral antigens.

## Perspective

Although never becoming an approved therapy outside the Chinese market the ONYX-015 (Sunway H-101) virus, the first oncolytic virus to undergo extensive clinical testing, clearly demonstrated therapeutic responses in at least a subset of patients treated. Researchers in the field have spent the last 15 years trying to enhance the activity and deliverability of these vectors so as to achieve more reliable and significant responses in the clinic. Although approaches to enhance the delivery of the vectors have met only limited success, recent clinical results with T-Vec and Pexa-Vec clearly show that significant improvements have been made in the anti-tumor activity of the vectors, especially when intratumoral delivery is employed. This has apparently been achieved through a combination of use of a faster replicating backbone and expression of an immune stimulating transgene. It is felt to be unlikely that significant additional advantage will be achieved through further enhancing replication potential without safety concerns being raised. The main avenue for further enhancing therapeutic potential may therefore be through careful enhancement of the interaction of the vectors with the host immune response. In this respect, it may be possible to learn from the advances made recently in the fields of vaccine development and cancer immunotherapy. However, it is also clear that simply activating immune stimulation will be unlikely to result in improved therapeutic activity, instead leading to reduced oncolytic activity through rapid clearance of the virus, possible with reduced induction of anti-tumor immunity. Instead, the most promising approaches look to redirect or subtly manipulate the immune response. This goal is complex and relies on inducing an increased recognition of weak tumor antigens with less targeting of typically much stronger viral antigens; increased CTL induction, with reduced humoral response; all while having minimal effects on viral oncolytic activity. However, recent pre-clinical data indicate that some major advances have been made in achieving these goals and there is renewed hope that next generation clinical vectors will significantly improve responses in a variety of cancer patients.

## Conflict of Interest Statement

The author declares that the research was conducted in the absence of any commercial or financial relationships that could be construed as a potential conflict of interest.
